# Association between HOMA-IR and Frailty among U.S. Middle-aged and Elderly Population

**DOI:** 10.1038/s41598-019-40902-1

**Published:** 2019-03-12

**Authors:** Po-Sen Peng, Tung-Wei Kao, Pi-Kai Chang, Wei-Liang Chen, Po-Jui Peng, Li-Wei Wu

**Affiliations:** 1 Division of Cardiology, Department of Internal Medicine, Zuoying Branch of Kaohsiung Armed Forces General Hospital, Kaohsiung, Taiwan Republic of China; 2 Division of Cardiology, Department of Internal Medicine, Tri-Service General Hospital; and School of Medicine, National Defense Medical Center, Taipei, Taiwan Republic of China; 3 Division of Family Medicine, Department of Family and Community Medicine, Tri-Service General Hospital; and School of Medicine, National Defense Medical Center, Taipei, Taiwan Republic of China; 4 Division of Geriatric Medicine, Department of Family and Community Medicine, Tri-Service General Hospital; and School of Medicine, National Defense Medical Center, Taipei, Taiwan Republic of China; 5 Division of Colon and Rectal Surgery, Department of Surgery, Tri-Service General Hospital; and School of Medicine, National Defense Medical Center, Taipei, Taiwan Republic of China; 6 Department of Psychiatry, Beitou Branch, Tri-Service General Hospital; and School of Medicine, National Defense Medical Center, Taipei, Taiwan Republic of China; 7 Graduate Institute of Medical Sciences, National Defense Medical Center, Taipei, Taiwan Republic of China

## Abstract

Previous literatures revealed that homeostasis model assessment-estimated insulin resistance (HOMA-IR) was one of the cardio-metabolic risk factors. This study was conducted to access the association between HOMA-IR and frailty in the United States of America (U.S.) middle-aged and elderly high-risk insulin-resistant population. In the National Health and Nutrition Examination Survey (NHANES III) from 1988 to 1994, the study included 3,893 participants. In order to exam the association between HOMA-IR and frailty in the middle-aged and elderly population through the regression model adjusted for multiple covariates, we divided the participants into middle aged group (Age <65 years) and elderly group (Age > = 65 years) in this study. Each group was then divided into tertiles depending on their HOMA-IR levels. Higher level of HOMA-IR was significantly associated with frailty in the elderly group, but this association was not seen in the middle-aged population. These results demonstrated that the HOMA-IR level can be a novel risk assessment of frailty in elderly high-risk insulin-resistant individuals (Age > = 65 years).

## Introduction

Frailty, a preventable geriatric syndrome, is a state of decline in physical function and decreased ability to deal with acute stress, which will lead to major adverse health events, including falls, disability, hospitalization, and death^1–4^. The prevalence of frailty is getting higher in the aging society^5^. Among individuals with diabetes and other chronic comorbidities, frailty is even more widespread. Due to its serious consequences, numerous studies aiming to identify biological markers for preventing the onset of frailty had been conducted and several human biomarkers, such as C-reactive protein (CRP), interleukin (IL)-6, tumor necrosis factor-alpha (TNF-α), transferrin, fibrinogen, serum total bilirubin levels, serum uric acid concentrations, serum 25(OH)D concentrations, serum triglycerides levels, etc., had been identified to be associated with frailty^6–11^.

Homeostasis model assessment-estimated insulin resistance (HOMA-IR) is a more convenient quantification tool for insulin resistance, compared with the gold standard hyperinsulinemic euglycemic clamp method. Several previous researches also used this tool to estimate the occurrence of frailty. Barzilay, J.I., *et al*.^9^ found that frailty syndrome was positively associated with IR, but the difference between middle-aged and elderly subgroup was still unknown. To further explore these questions, we enrolled high-risk insulin-resistant population and then divided the participants into middle-aged (age <65 years) and elderly groups (age >=65 years) and investigated the relationship between HOMA-IR and frailty from the third National Health and Nutrition Evaluation Survey (NHANES III) data in the United States of America (U.S.).

## Results

A total of 3,893 adults in the NHANES III database were included in this study. Table 1 lists the clinical characteristics of frailty and non-frailty group within the middle aged (Age <65 years) and elderly population (Age >=65 years). Participants in middle aged (Age <65 years) frailty group tended to have higher body weight, higher waist circumference and body mass index (BMI), higher serum C-reactive protein, and higher insulin and HOMA-IR levels than those in non-frailty group. Fewer ideal physical activities were found in the frailty group.

**Table 1:** Participant characteristics of frailty and non-frailty in middle-aged and elderly adults.

Variables	Middle aged (Age <65) N = 1130		Total N = 1130	P-value	Elderly group (Age > = 65) N = 2763		Total N = 2763	P-value
	Non-frailty group N = 1092	Frailty group N = 38			Non-frailty group N = 2598	Frailty group N = 165		
**Continuous Variables**								
Age (years), mean (SD)	62.52 (1.72)	62.47 (1.72)	62.52 (1.72)	0.880	75.17 (6.44)	77.22 (6.68)	75.29 (6.47)	<0.001
Weight (kg) (2 months and over)	76.58 (15.75)	85.61 (23.22)	76.88 (16.13)	0.001	71.88 (15.08)	70.65 (16.85)	71.81 (15.19)	0.311
Waist circumference (cm) (2 + years)	98.15 (12.42)	107.35 (16.10)	98.46 (12.66)	<0.001	96.57 (12.27)	98.30 (14.09)	96.67 (12.39)	0.082
BMI (kg/m^2^)	27.65 (4.84)	32.33 (8.99)	27.81 (5.10)	<0.001	26.55 (4.78)	27.86 (6.29)	26.63 (4.89)	0.001
Systolic BP (mmHg), mean (SD)	134.57 (21.20)	136.92 (20.29)	134.65 (21.17)	0.507	144.10 (23.99)	144.93 (25.17)	144.15 (24.05)	0.672
Diastolic BP (mmHg), mean (SD)	73.74 (13.04)	75.62 (11.92)	73.81 (13.00)	0.388	71.97 (14.27)	69.48 (15.64)	71.82 (14.36)	0.034
Serum triglycerides (mg/dL)	167.59 (113.11)	168.03 (115.37)	167.60 (113.13)	0.981	153.58 (91.02)	163.28 (124.93)	154.16 (93.40)	0.196
Serum cholesterol (mg/dL), mean (SD)	226.44 (44.92)	225.61 (51.31)	226.41 (45.13)	0.911	221.58 (43.82)	221.72 (45.09)	221.58 (43.89)	0.969
Serum LDL-cholesterol (mg/dL), mean (SD)	144.91 (41.69)	142.39 (46.22)	144.82 (41.81)	0.802	139.08 (38.27)	134.26 (36.86)	138.84 (38.20)	0.337
Serum HDL-cholesterol (mg/dL), mean (SD)	50.98 (16.31)	55.66 (17.90)	51.14 (16.38)	0.084	51.51 (16.35)	56.49 (18.91)	51.81 (16.55)	<0.001
Serum C-reactive protein (mg/dL), mean (SD)	0.57 (1.17)	1.14 (1.28)	0.58 (1.18)	0.003	0.55 (0.90)	0.51 (0.51)	0.54 (0.89)	0.581
Serum total bilirubin (umol/L), mean (SD)	0.58 (0.32)	0.53 (0.43)	0.58 (0.33)	0.344	0.60 (0.28)	0.54 (0.29)	0.60 (0.28)	0.004
Serum uric acid (mg/dL)	5.59 (1.45)	5.74 (1.96)	5.59 (1.47)	0.529	5.73 (1.55)	5.89 (1.66)	5.74 (1.56)	0.191
Serum glucose (mg/dL), mean (SD)	105.25 (34.51)	108.29 (32.22)	105.36 (34.43)	0.593	104.79 (31.44)	107.53 (36.60)	104.95 (31.77)	0.283
Serum total protein (g/dL), mean (SD)	7.37 (0.47)	7.47 (0.54)	7.38 (0.47)	0.206	7.29 (0.48)	7.40 (0.55)	7.30 (0.48)	0.004
Aspartate aminotransferase (U/L), mean (SD)	22.30 (12.47)	20.32 (8.15)	22.23 (12.35)	0.331	21.51 (10.41)	20.85 (7.92)	21.47 (10.28)	0.420
Alanine aminotransferase (U/L), mean (SD)	16.52 (10.52)	14.47 (7.24)	16.45 (10.43)	0.235	13.51 (9.40)	12.70 (7.84)	13.46 (9.31)	0.283
Insulin (uU/mL), mean (SE)	12.20 (7.29)	16.85 (12.54)	12.36 (7.57)	<0.001	11.26 (7.29)	13.77 (8.74)	11.41 (7.41)	<0.001
HOMA-IR, mean (SD)	3.35 (2.56)	4.73 (3.66)	3.39 (2.61)	0.001	3.07 (2.40)	3.89 (3.20)	3.12 (2.47)	<0.001
**Categorical Variables**								
Non-Hispanic white, N(%)	482 (44.1)	13 (34.2)	507 (43.8)	0.120	1748 (67.3)	75 (45.5)	1823 (66.0)	<0.001
Stroke, N (%)	38 (3.5)	3 (7.9)	41 (3.6)	0.153	168 (6.5)	31 (18.8)	199 (7.2)	<0.001
DM, N (%)	131 (12)	4 (10.5)	135 (11.9)	0.929	300 (11.5)	40 (24.2)	340 (12.3)	<0.001
Smoker, N (%)	174 (15.9)	5 (13.2)	179 (15.8)	0.822	470 (18.1)	18 (10.9)	488 (17.7)	0.020
Physical activity, ideal, N (%)	329 (30.1)	10 (26.3)	339 (30.0)	<0.001	963 (37.1)	28 (17.0)	991 (35.9)	<0.001

Abbreviation: N, number; SD, standard deviation; BMI, body mass index; SBP, systolic blood pressure; LDL, low-density lipoprotein; ALT, alanine aminotransferase; CRP, C-reactive protein.

Among the elderly population (Age >=65 years), age, BMI, serum high-density lipoprotein (HDL) levels, serum total protein levels, serum insulin and HOMA-IR levels were significantly greater in the frailty group. However, diastolic blood pressure, serum total bilirubin levels were lower in the frailty group. There were fewer non-Hispanic white, fewer smoker, fewer physical activities, but more stroke, and diabetes mellitus in the frailty group.

Positive associations between HOMA-IR tertiles and the risk of frailty were observed in elderly adults (Age > = 65 years) as shown in Table 2. The p-values for the trend in the elderly group in model 1, model 2, model 3, and model 4 were 0.001, 0.009, 0.060, and 0.056, respectively.

**Table 2:** Associations between the frailty and tertiles of HOMA-IR level in middle-aged and elderly adults.

Models^a^	Tertiles of HOMA-IR	*β*^b^ (95% CI)	*P* value	*P* for trend	*β*^b^ (95% CI)	*P* value	*P* for trend
		Middle-aged group (Age <65 years)			Elderly group (Age > = 65 years)		
Model 1	T2 v.s. T1	0.58 (0.17–2.07)	0.403	0.081	1.93 (1.14–3.28)	0.015	0.001
	T3 v.s. T1	1.87 (0.73–4.79)	0.193		2.72 (1.63–4.55)	<0.001	
Model 2	T2 v.s. T1	0.36 (0.10–1.30)	0.119	0.296	1.70 (0.99–2.93)	0.056	0.009
	T3 v.s. T1	0.65 (0.23–1.84)	0.415		2.42 (1.37–4.29)	0.002	
Model 3	T2 v.s. T1	0.38 (0.10–1.40)	0.146	0.332	1.61 (0.93–2.80)	0.090	0.060
	T3 v.s. T1	0.73 (0.22–2.37)	0.597		2.12 (1.14–3.95)	0.018	
Model 4	T2 v.s. T1	0.37 (0.10–1.37)	0.136	0.304	1.62 (0.93–2.82)	0.086	0.056
	T3 v.s. T1	0.75 (0.23–2.49)	0.643		2.14 (1.15–4.00)	0.016	

^a^Adjusted covariates: Model 1 = Unadjusted.

Model 2 = Model 1 + age, sex, race/ethnicity, BMI, systolic blood pressure.

Model 3 = Model 2 + serum fasting glucose, serum uric acid, serum C-reactive protein.

Model 4 = Model 3 + history of stroke, smoking, physical activity.

^b^β coefficients was interpreted as change of frailty for each increase in the HOMA-IR level.

However, the association between HOMA-IR tertiles and the risk of frailty in the middle-aged (Age <65 years) group all showed statistically insignificant. The p-values for the trend among middle-aged group were 0.081 in model 1 and 0.296, 0.332, 0.304 in model 2, model 3, and model 4, respectively.

With regard to frailty components associated with HOMA-IR tertiles (Table 3), there were positive associations between HOMA-IR tertiles and the frailty components of weakness, exhaustion, and low physical activity in elderly adults (Age >=65 years). Concerning frailty components associated with HOMA-IR tertiles in the middle aged group (Age <65 years), only low physical activity was positively associated with HOMA-IR level.

**Table 3:** Associations between the frailty components and tertiles of HOMA-IR level in middle-aged and elderly adults.

Models^a^	Tertiles of HOMA-IR	β^b^ (95% CI)	*P* value	*P* for trend	β^b^ (95% CI)	*P* value	*P* for trend
		Middle-aged group (Age <65 years)			Elderly group (Age > = 65 years)		
Slow walking							
Model 1	T2 v.s. T1	0.76 (0.35–1.64)	0.479	0.034	1.09 (0.85–1.41)	0.499	0.277
	T3 v.s. T1	1.70 (0.91–3.19)	0.097		1.23 (0.95–1.58)	0.113	
Model 2	T2 v.s. T1	0.58 (0.26–1.27)	0.172	0.269	1.02 (0.78–1.33)	0.875	0.199
	T3 v.s. T1	0.97 (0.49–1.94)	0.935		1.25 (0.94–1.67)	0.125	
Model 3	T2 v.s. T1	0.57 (0.26–1.26)	0.165	0.304	0.97 (0.74–1.27)	0.081	0.843
	T3 v.s. T1	0.91 (0.42–1.98)	0.803		1.05 (0.76–1.45)	0.780	
Model 4	T2 v.s. T1	0.54 (0.24–1.21)	0.135	0.271	0.96 (0.74–1.26)	0.791	0.850
	T3 v.s. T1	0.86 (0.39–1.90)	0.713		1.04 (0.95–1.44)	0.810	
Weakness							
Model 1	T2 v.s. T1	1.16 (0.88–1.52)	0.291	<0.001	1.11 (0.89–1.38)	0.363	0.001
	T3 v.s. T1	1.63(1.27–2.09)	<0.001		1.47 (1.19–1.82)	<0.001	
Model 2	T2 v.s. T1	1.03 (0.78–1.37)	0.842	0.053	1.10 (0.88–1.39)	0.396	<0.001
	T3 v.s. T1	1.35 (1.01–1.80)	0.040		1.64 (1.29–2.10)	<0.001	
Model 3	T2 v.s. T1	1.03 (0.78–1.38)	0.820	0.058	1.07 (0.84–1.35)	0.590	0.002
	T3 v.s. T1	1.38 (1.01–1.88)	0.042		1.53 (1.17–2.01)	0.002	
Model 4	T2 v.s. T1	1.04 (0.78–1.38)	0.789	0.077	1.06 (0.84–1.34)	0.620	0.003
	T3 v.s. T1	1.36 (1.00–1.86)	0.050		1.52 (1.15–1.99)	0.003	
Exhaustion							
Model 1	T2 v.s. T1	1.52 (0.88–2.64)	0.136	0.256	1.84 (1.18–2.88)	0.007	<0.001
	T3 v.s. T1	1.49 (0.87–2.56)	0.147		2.43 (1.58–3.75)	<0.001	
Model 2	T2 v.s. T1	1.27 (0.72–2.26)	0.410	0.668	1.60 (1.01–2.53)	0.044	0.010
	T3 v.s. T1	1.08 (0.58–2.01)	0.813		2.11 (1.30–3.41)	0.002	
Model 3	T2 v.s. T1	1.24 (0.70–2.21)	0.463	0.685	1.61 (1.01–2.57)	0.045	0.026
	T3 v.s. T1	1.03 (0.53–2.01)	0.927		2.06 (1.22–3.50)	0.007	
Model 4	T2 v.s. T1	1.25 (0.70–2.23)	0.445	0.648	1.62 (1.02–2.58)	0.043	0.026
	T3 v.s. T1	1.02 (0.52–1.99)	0.953		2.07 (1.22–3.50)	0.007	
Low physical activity							
Model 1	T2 v.s. T1	1.14 (0.94–1.38)	0.191	<0.001	1.58 (1.15–2.16)	0.005	<0.001
	T3 v.s. T1	1.87 (1.57–2.22)	<0.001		2.36 (1.74–3.18)	<0.001	
Model 2	T2 v.s. T1	1.03(0.84–1.25)	0.799	<0.001	1.45 (1.05–2.01)	0.025	<0.001
	T3 v.s. T1	1.48 (1.20–1.81)	<0.001		2.16 (1.54–3.03)	<0.001	
Model 3	T2 v.s. T1	1.01 (0.83–1.24)	0.905	0.002	1.35 (0.97–1.87)	0.077	0.012
	T3 v.s. T1	1.39 (1.11–1.74)	0.004		1.75 (1.21–2.54)	0.003	
Model 4	T2 v.s. T1	1.01 (0.83–1.24)	0.908	0.003	1.34 (0.96–1.87)	0.082	0.012
	T3 v.s. T1	1.39 (1.11–1.74)	0.004		1.75 (1.21–2.53)	0.003	
Low body weight							
Model 1	T2 v.s. T1	0.36 (0.25–0.53)	<0.001	<0.001	0.23 (0.15–0.37)	<0.001	<0.001
	T3 v.s. T1	0.09 (0.05–0.19)	<0.001		0.07 (0.03–0.16)	<0.001	
Model 2	T2 v.s. T1	1.41 (0.94–2.12)	0.097	0.155	1.60 (0.95–2.69)	0.077	0.147
	T3 v.s. T1	1.62 (0.77–3.42)	0.202		0.81 (0.35–1.88)	0.620	
Model 3	T2 v.s. T1	1.44 (0.94–2.21)	0.096	0.212	1.27 (0.73–2.20)	0.397	0.107
	T3 v.s. T1	1.61 (0.64–4.03)	0.310		0.42 (0.14–1.21)	0.107	
Model 4	T2 v.s. T1	1.46 (0.95–2.24)	0.086	0.207	1.26 (0.73–2.18)	0.415	0.109
	T3 v.s. T1	1.53 (0.61–3.82)	0.368		0.41 (0.14–1.21)	0.106	

^a^Adjusted covariates: Model 1 = Unadjusted.

Model 2 = Model 1 + age, sex, race/ethnicity, BMI, systolic blood pressure.

Model 3 = Model 2 + serum fasting glucose, serum uric acid, serum C-reactive protein.

Model 4 = Model 3 + history of stroke, smoking, physical activity.

^b^β coefficients was interpreted as change of frailty components for each increase in the HOMA-IR level.

Abbreviation: ADL, activities of daily living; GPA, general physical activities; IADL, instrumental activities of daily living; LEM, lower extremity mobility; LSA, leisure and social activities.

## Discussion

In the non-institutionalized U.S. high-risk insulin-resistant population, we investigated the relationship between HOMA-IR level and frailty. No previous study had comprehensively evaluated the link between HOMA-IR level and frailty in US middle-aged and elderly participants. The most remarkable finding was a positive correlation between HOMA-IR level and frailty in elderly adults. However, we did not find such association among the middle-aged group.

Frailty is a dynamic state affecting an individual who experiences accelerated decline in several domains of homeostatic mechanisms (physical, psychological, and social)^12^. One previous study showed the predictive value of HOMA-IR on habitual gait speed, one of the domains of frailty^13^. Furthermore, Joshua I. Barzilay *et al*.^9^ also reported that frailty syndrome was positively associated with IR, but the difference between middle-aged and elderly population was not analyzed.

The physiopathology of frailty syndrome is multifactorial and includes nutritional, physical and hormonal elements. The first possible mechanism is that chronic low-grade inflammation, and oxidative status has an effect on frailty syndrome. Darvin, K. *et al*. found that frailty adults had higher plasma concentrations in inflammatory markers such as transferrin, fibrinogen, and IL-6^11^. A meta-analysis also demonstrated that in frail participants dwelling in the community, the CRP and IL-6 levels were significantly higher than the non-frail population^14^. Chronic inflammation leads to increased IR and muscle dysfunction, subsequently results in frailty syndrome in elderly adults^15^.

Another possible mechanism is that the decline in insulin sensitivity causes a defect in muscle mass catabolism and muscle quality, resulting in sarcopenia and loss of strength^1,16–18^. Lower muscle mass also results in poorer blood glucose control through lower peripheral glucose uptake by skeletal muscle and this will cause hyperinsulinemia status and insulin resistance^16^. The crosstalk interaction increases frailty incidence.

The alternative explanation may be the endocrine theory. Muscle mass is thought to be a metabolic tissue and endocrine organ, and several endocrines are released from muscle mass, called myokines. These myokines can increase insulin sensitivity, elevate mitochondrial activity and modulate body composition, such as playing an anabolic effect on skeletal muscle, and decreasing muscle protein degradation^19^. The increase of IR causes loss of muscle mass, resulting in the defect of myokines. Accordingly, the virtuous cycle of IR and sarcopenia may subsequently result in impaired body energy regulation and performance, and increase the frailty incidence. Other studies^20,21^ had also highlighted that three major circulating hormones were decreased in ageing body, including insulin-like growth factor-1(IGF-1), sex hormone, dehydroepiandrosterone and dehydroepiandrosterone sulfate production. IGH-1 can improve insulin sensitivity mediated through skeletal muscle and the deficiency of the IGF-1 actions cause to insulin resistance^22,23^. These hormones changes are considered to cause frailty in elderly people.

A cross-sectional study researched the relationship between cumulative physiological dysfunction in six different systems and frailty (hormonal, micronutrients, haematological, inflammatory, adiposity, and neuromuscular system), which reported three or more systems dysfunction were able to predict frailty, independent of age and comorbidity^24^. The elderly people may have poorer nutritional status than younger population and poor nutrition can also be a mediating factor to frailty^3^. Furthermore, anemia is more frequently observed in aging population and the etiologies are often associated with the presence of chronic diseases through several pathway^25^. Anemia can contribute to functional decline by means of the restriction of oxygen delivery to muscle, and some vital organ, such as brain. Anemia can be a risk factor to the frailty syndrome and even be an independent risk factor in community-dwelling elderly women^26–28^. The elderly people who have overlapping comorbidities are more frequent than middle-aged and the contribution of subclinical diseases may be associated to increase the incidence of frailty^29^.

Several cross interactions between insulin resistance, certain systemic diseases, and frailty deserve to be mentioned. Insulin resistance is positively associated with the development of heart failure^30,31^ independent of coronary artery disease^32^. In advanced stage of heart failure, the cardiomyocytes are reversed to fetal form and the use of glucose as a fuel is increased instead of free fatty acids^33^. Impairment in glucose use and higher fasting insulin level during insulin resistance status will result in heart failure due to energy starvation. Several evidence also implicated that insulin resistance accompanied with hyperinsulinemia was a predictor of cancers^34,35^. Insulin has effects on cell proliferation, and may promote cancer through this mechanism^36^. In addition, loss of lean muscle mass and alteration in body adipose tissue are frequently found in cancer cachexia. The cachexia associated metabolic derangements will promote pro-inflammatory status and insulin resistance^37^. Further evidence also showed that insulin resistance was an independent and significant risk factor for ischemic stroke through enhancing platelet aggregation and atherosclerosis change^38–40^. In our study, we had excluded the participants who had congestive heart failure, cancer, and asthma, because these diseases may directly contribute to frailty, instead of owing to insulin resistance pathway.

Some characteristic differences between non-frailty and frailty group in the elderly population showed statistically significant in our study. The lower total bilirubin level and higher HDL level in the frailty group of elderly population were worthy of being discussed. The pathophysiology of frailty is a complex interaction involved oxidative stress and inflammation^41^. Emerging evidence supported the anti-inflammatory and antioxidant effects of serum total bilirubin^42,43^ and reported that serum total bilirubin was inversely associated with likelihood of functional dependence in elderly adults^44^. Functional impairment and frailty are closely associated with lower HDL level^45^. In contrast to previous evidence, the HDL level was higher in our frailty group of elderly population. The possible explanation was the benefits of higher HDL level may be overtaken by other comorbidities in our frailty group of elderly population.

This study had several potential limitations. Firstly, the sample size of middle aged individuals was relatively smaller to elderly group, which may have caused some biases. We will continue to expand the sample size of middle aged individuals to study the relationship of HOMA-IR and frailty. Secondly, the study using NHANES III database was a cross-sectional study, that cannot determine the causality between HOMA-IR level and increased risk of frailty. Only prospective study can overcome this limitation. Thirdly, there may be some comorbidities in participants that limited their human functions and some drugs which can contribute to frailty and insulin resistance we didn’t adjust. Although we had adjusted multiple potential confounding variables, it’s difficult to adjust comprehensively due to limited by data available in the NHANES. Fourthly, recall bias is the inherited limitation of cross-sectional studies, and may not represent real conditions among the participants.

In conclusion, this study demonstrated that in the U.S. high-risk insulin-resistant population, HOMA-IR level can have good predictive ability of frailty in elderly population.

## Methods

### Study population

The data were obtained from NHANES III of the U.S. non-institutionalized population, from 1988 to 1994, who underwent comprehensive household interview and health examination performed at a mobile examination center. A total of 3,893 participants with aged 40–90 years were enrolled in this cross-sectional study. The examined data in NHANES III included several contents: demographic data, extensive household interview (information on age, sex, race, and medical history), physical examination, blood sampling, anthropometric measurement, and body-composition assessment. Written informed consents of all participants were obtained before beginning.

### HOMA-IR Level Tertiles-based Subgroups

HOMA-IR index was estimated using the following formula: HOMA-IR = [fasting serum insulin (mU/L) × fasting plasma glucose (mmol/L)]/22.5^46^. We separated all participants into two groups based on age: age <65 years (middle-aged group), age >=65 years (elderly group). Each group was then categorized into three tertiles according to their HOMA-IR level. The tertiles were as follows: T1 (0.34–1.75), T2 (1.76–3.09), T3 (3.10–16.17) in the middle-aged group and T1 (0.30–1.80), T2 (1.81–3.08), T3 (3.09–16.23) in the elderly group.

### Follow-up data on frailty

Frailty was defined based on the previously validated frailty criteria originally reported by Fried *et al*.^3^. We followed the definition and modified the criteria for application to NHANES III data. The main modifications of frailty were that the nutritional status was based on low BMI instead of weight loss in the preceding months, and weakness/ Low physical activity were self-reported, not measured by direct assessment of grip strength and physical activity.

The five modified domains of frailty criteria were as follows: (a) slow walking was defined as in the 8 foot walking speed test, participants within the worst quintile adjusted for sex; (b) weakness, defined as present if participants were asked the question “How much difficulty you have while lifting or carrying something as heavy as 10 pounds“, they responded this question as some difficulty, much difficulty, or unable to do it; (c) exhaustion, defined as present if participants had the answer as some difficulty, much difficulty, or unable to do it to the question “How much difficulty you have while walking from one room to the other on the same level?”; (d) low physical activity, defined as present if participants answered less active to the question “When compared to most men/women of your age, would you say that you are more active, less active or about the same?”; (e) BMI less than 18 kg/m^2^ was defined as low body weight.

Participants who met 3 or more of 5 domains were considered as frailty. We dichotomized all participants into “frailty group” (3 or more frailty criteria) and “non-frailty group” (0–2 frailty criteria) for the purposes of our analyses.

### Covariates

Questionnaire information that may act as potential confounders independently to the outcome included age, sex, race/ethnicity, medical conditions (stroke, type 2 diabetes mellitus), smoking status, and physical activity. Self-reported race/ethnicity was separated into non-Hispanic white and others. Smoking status was obtained during the interview and was categorized into smokers and non-smokers. Chronic disease, such as stroke was ascertained from self-reports. Diabetes mellitus was defined if they had been diagnosed by a doctor, or using anti-diabetic drugs (insulin injections and/or oral anti-diabetic agents), or random serum glucose level ≥200 mg/dL, or the fasting glucose level was ≥126 mg/dL.

Systolic and diastolic blood pressure were measured three times at 1–2 min intervals and to get the average of these readings after the participant seated for 5 min using a mercury sphygmomanometer with an appropriate sized cuff. Weight and height were measured in standardized conditions, and were used to calculate BMI as weight in kilogram divided by squared height in meters. The serum biochemical profiles analyses were performed in standard protocols and the documented accuracy was approved by the Centre for Disease Control and Prevention (CDC). All detailed information about standardized protocols was available on the NHANES website.

The following physical activity questionnaires were collected: which and how frequently they got involved in leisure time physical activities during the past month. The physical activities included riding a bicycle, swimming, jogging or running (≥1 mile), weight training and yard work. Metabolic equivalent tasks (METs), the ratio of the metabolic rate during the activity to the basal metabolic rate were used as a means of expressing the energy cost of physical activities among persons of different weight. We measured the energy expenditure in physical activity using the summary of METs and participants were further classified as ideal and non-ideal group in Table 1 in this article. The ideal group was defined as that they got involved in any physical activity 5 or more times per week with the intensity of 3 to 5.9 METs per times or in any physical activity 3.0 or more times per week with the intensity of 6 or more METs per times.

### Statistical analysis

Statistical Product and Service Solutions (SPSS) version 18 (SPSS, Inc., Chicago, IL, USA) were used for all data management. Characteristics of the study population were calculated according to the survey design. Continuous variables were presented as mean value and standard deviations (SD), and qualitative variables were presented as numbers with percentages. Differences in continuous data were compared by the independent t-test, and comparisons of categorical variables were conducted by Chi-square test. Multivariable logistic regression models were used to calculate the odds ratios of frailty based on the tertiles of HOMA-IR in middle aged and elderly population. The significance tests were two-sided and p-values < 0.05 were considered to indicate statistical significance. We used the HOMA-IR level as a continuous variable to examine the associations between an increase of HOMA-IR level and frailty, and p-values for the trend were analyzed. The model-adjusted method was used to adjust the potential confounding effects of covariates: in model 1, there were no variables adjusted; Model 2 was further adjusted for age, sex, race/ethnicity, BMI, systolic blood pressure; Model 3 consisted of Model 2 and was additionally adjusted for serum fasting glucose, serum uric acid, serum C-reactive protein. Model 4 was additionally adjusted for history of stroke, smoking, physical activity.

### Ethics statement

The National Center for Health Statistics Institutional Review Board (IRB) approved the NHANES study protocol before beginning. Before data collection procedures and NHANES health examinations, informed consents had been obtained from all participants. All methods in this study were performed in the light of the relevant guidelines and regulations.
